# Calcium Rescues *Streptococcus pneumoniae* D39 Δ*mntE* Manganese-Sensitive Growth Phenotype

**DOI:** 10.3390/microorganisms12091810

**Published:** 2024-09-01

**Authors:** Reuben Opoku, Edgar Carrasco, Nicholas R. De Lay, Julia E. Martin

**Affiliations:** 1 Department of Biological Sciences, Idaho State University, Pocatello, ID 83209, USA; 2 Department of Microbiology and Molecular Genetics, McGovern Medical School, University of Texas Health Science Center, Houston, TX 77030, USA; 3 MD Anderson Cancer Center UT Health Graduate School of Biomedical Sciences, University of Texas Health Science Center, Houston, TX 77030, USA

**Keywords:** streptococcus, manganese, calcium, metal homeostasis, bacterial capsule, biofilm

## Abstract

Calcium (Ca^2+^) functions as a universal signal messenger in eukaryotes but in bacteria, the physiological roles for Ca^2+^ are limited. Here, we examine the role of Ca^2+^ in *Streptococcus pneumoniae* during manganese (Mn^2+^) intoxication. *S. pneumoniae mntE* mutants, lacking the Mn^2+^ efflux transporter, exhibit impaired growth due to accumulation of Mn^2+^ when exposed to elevated exogenous Mn^2+^. This Mn^2+^-sensitive growth defect is restored to wild-type growth level by exogenous Ca^2+^, in a Ca^2+^-dependent manner. Despite growth restoration of the *mntE* mutant to wild-type levels, cellular Mn^2+^ remains elevated in this strain. Bacterial capsule production is also increased for the *mntE* mutant, resulting in reduced adherence capacity to surfaces and poor biofilm formation, which is consistent with it experiencing Mn^2+^ intoxication. Ca^2+^ presence did not significantly impact bacterial capsule production or biofilm formation. Further analysis of the cell morphology demonstrates that Ca^2+^ contributes to cell division and reduces cell chain lengths. Together, these data describe the first role of Ca in *S. pneumoniae* that has potential implications in bacterial virulence since Ca affects cell division and likely Mn^2+^-associated cellular processes.

## 1. Introduction

Calcium (Ca^2+^) is an essential metal micronutrient present in the eukaryotic host environment that plays both structural and regulatory roles in a myriad of cellular processes ranging from cell division, transport, to host immune defenses against pathogens [1,2]. However, in bacteria, the physiological roles for Ca^2+^ remain elusive. A growing body of evidence indicates that Ca^2+^ may serve as a signaling molecule in regulating bacterial cellular processes from cell division, transport, stress response, competence, to lifestyle switches that include sporulation and biofilm formation [3,4,5,6,7].

More than three-quarters of all human microbial infections are due to biofilms [8,9]. These biofilms are not only associated with medical devices but also with the mucosa of the gastrointestinal and respiratory tracts. The concentration of Ca^2+^ in respiratory-associated human body fluids is 1–5 mM [10] and is up to 10,000 times higher than host cell cytosolic Ca^2+^ concentrations [11,12]. Bacterial pathogens can induce changes in host cell cytosolic Ca^2+^ concentrations via bacterial surface-associated protein or toxin interactions with host cells [13,14,15]. Such alterations in host Ca^2+^ levels have been shown to facilitate bacterial adherence, enhance cell aggregation, and increase the release of extracellular DNA, all of which contribute to strengthening biofilms formed by *Streptococcus* strains [16,17]. We note here that adherence is necessary for initial *Streptococcus pneumoniae* colonization in the nasopharynx of the human upper respiratory tract, and that biofilm formation is important for *S. pneumoniae* persistence and survival within the host [18].

In *S. pneumoniae*, Ca^2+^ is an obligatory metal micronutrient required at 150 µM for viability in laboratory medium [19]. Increasing the Ca^2+^ concentration to ≥1 mM induces two different cell states: genetic competence during exponential growth to take up DNA and cell lysis when entering the stationary phase of growth to release DNA [19,20]. It is important to note that *S. pneumoniae* is an extracellular pathogen and its natural environment is human body fluids in which the Ca^2+^ concentration as described above is ≥1 mM, the concentration at which *S. pneumoniae* competence and lysis are obtained under laboratory conditions [19].

As with host cells, the intracellular Ca^2+^ concentration range in bacteria is 80–100 nM [21,22], indicating that bacteria like their hosts also regulate Ca^2+^ storage and flux across their membranes. It is also imperative that bacterial pathogens sense Ca^2+^ levels, since Ca^2+^ concentrations differ among host infection sites and fluctuate during bacterial disease progression. To date, several Ca^2+^-sensing and Ca^2+^-dependent regulatory systems, in addition to homologs of eukaryotic Ca^2+^-leak channel and pumps, have been identified among bacteria that function in virulence and pathogenesis [10]. Much remains to be learned for Ca^2+^ homeostasis in many bacterial pathogens, including *S. pneumoniae*.

In comparison to Ca^2+^, Mn^2+^ is well recognized to serve a versatile role in bacteria at the host–pathogen interface, aiding in bacterial virulence, and is both essential for bacterial viability and toxic in excess [23]. As such, many bacteria, including *Streptococcus pneumoniae*, employ complex regulatory networks to control Mn^2+^ homeostatic levels that optimize colonization and growth within the host. Like Ca^2+^, the Mn^2+^ concentration varies across body sites and fluid ranging from 650 nM in the nasopharynx where *S. pneumoniae* commonly colonizes to 1100 nM Mn^2+^ in the lung; the blood contains 400–700 nM Mn^2+^ [24]. Note that these concentrations reflect total Mn^2+^ and do not represent the bioavailable free Mn^2+^ that bacteria like *S. pneumoniae* physically experience and use during pathogenesis in the host. As such, the topic of when and where do pathogenic bacteria experience Mn^2+^ intoxication remains obscure. It is hypothesized that Mn^2+^ levels are not stagnant in the host, and that *S. pneumoniae* must be equipped to quickly adapt to constant metal ion fluctuations.

*S. pneumoniae* acquires Mn^2+^ via the Mn^2+^-specific ABC-type importer PsaBCA, which is negatively regulated by the Mn^2+^-dependent DtxR family transcriptional corepressor PsaR [25,26]. Excess cellular Mn^2+^ is exported out of the cell by the Mn^2+^-specific cation diffusion facilitator (CDF) transporter MntE to prevent Mn^2+^ intoxication [27]. An additional metal-dependent P_1B_-type ATPase efflux transporter MgtA (formerly CaxP) is shown to function as a failsafe when cellular Mn^2+^ reaches ≥100 µM [28]. Previous characterization of the regulation of *mgtA* expression by a 5′ Mn/Ca^2+^-sensing *yybP-ykoY* family riboswitch revealed a potential intersectional role between Ca^2+^ and Mn^2+^ [28], prompting deeper examination of the Ca^2+^ impact on *S. pneumoniae* growth during Mn^2+^ stress.

This study is the first step in uncovering such cross-sectional roles for Ca^2+^ in Mn^2+^ physiological processes in *S. pneumoniae* by delving into the molecular complexities surrounding the cellular metal ion composition and its effect on bacterial virulence components.

## 2. Materials and Methods

### 2.1. Bacterial Growth Conditions

Brain heart infusion (BHI) and Todd Hewitt (TH) broth were of standard composition (BD Biosciences, San Jose, CA, USA) and prepared using ultrapure water (≈17.1 Ω). Both BHI and TH are general-purpose rich media for cultivation of fastidious microorganisms, including *Streptococcus* strains. Bacterial strains used in this study are listed in Table S1. All *S. pneumoniae* strains were grown in BHI or TH broth at 37 °C in a 5% CO_2_ atmosphere. Briefly, *S. pneumoniae* bacterial frozen stocks were inoculated into broth, serially diluted, and propagated overnight. The next morning, exponentially growing cultures were diluted to 0.005 OD_620_ in prewarmed broth supplemented with MnCl_2_ and CaCl_2_ as indicated, and growth was monitored over time at OD_620_.

All *Escherichia coli* and *Bacillus subtilis* strains were grown in BHI broth aerobically at 37 °C with vigorous shaking. Briefly, single colonies were inoculated into broth and propagated overnight. The next morning, overnight cultures were diluted to 0.01 OD_620_ in prewarmed broth. After approximately four generations of growth, exponentially growing cells were diluted to 0.005 OD_620_ into fresh prewarmed broth supplemented with MnCl_2_ and CaCl_2_ as indicated, and growth was monitored over time at OD_620_.

### 2.2. Dilution Drop Test

*S. pneumoniae* cultures were grown in BHI broth to approximately 0.40 OD_620_, then serially diluted 10-fold to 10^−5^ in BHI broth. An aliquot of 5 µL of each dilution was spotted onto BHI agar plates containing 250 U/mL of filtered bovine liver catalase (Worthington Biochemical, Lakewood, NJ, USA) and varying concentrations of MnCl_2_ and CaCl_2_ as indicated [29]. All plates were incubated at 37 °C in a 5% CO_2_ atmosphere for 24 h and documented photographically.

### 2.3. Metal Quantification

The total cell-associated metal ions for Mn^2+^, Fe^2+^, Zn^2+^, Cu^2+^, Mg^2+^, and Ca^2+^ were quantified from *S. pneumoniae* cultured for 3.5 h in BHI broth with MnCl_2_ and CaCl_2_ as indicated using slightly modified standard protocol previously described [30,31]. Briefly, cells (200 mL) were harvested by centrifugation at 4 °C, suspended in 1/200 volume of original culture with cold phosphate-buffered saline (PBS) pH 7.4, 2 mM EDTA, and washed twice with cold metal-free PBS (10 g/L chelex-100 resin was used to remove metals) pH 7.4 before storing at -80 °C. Metal quantifications of inactivated cell pellets were determined by the Center for Applied Isotope Studies (University of Georgia, Athens) using a Perkin Elmer 8300 ICP-OES (Perkin Elmer, Shelton, CT, USA). Metal quantifications were normalized to wet cell weight (wcw).

### 2.4. Uronic Acid Assay

*S. pneumoniae* was grown for 3.5 h in BHI broth with MnCl_2_ and CaCl_2_ as indicated prior to determining uronic acid concentrations using a standard protocol previously described [29,32]. Briefly, 10 mL culture was harvested by centrifugation after approximately 3.5 h of growth in BHI with MnCl_2_ or CaCl_2_ as indicated. Cell pellets were suspended in 1/20 the original culture volume with 150 mM Tris-HCl (pH 7.0)/1 mM MgSO_4_, incubated with 0.1% deoxycholate, and subjected to enzymatic digestion of the cell wall (100 U mutanolysin), nucleic acids (0.1 mg/mL DNase and 0.1 mg/mL RNase), and proteins (0.1 mg/mL proteinase K). The supernatant was collected after centrifugation, mixed with a 98% (*v*/*v*) sulfuric acid–12.5 mM tetraborate solution, boiled, cooled on ice, and mixed with 0.15% (*w*/*v*) 3-phenylphenol in 0.5% NaOH. The absorbance was read at 520 nm at room temperature within 5 min. Samples incubated with 0.5% NaOH served as the background, and measurements were subtracted out prior to normalization to total protein determined using the DC protein assay (Bio-Rad, Hercules, CA, USA).

### 2.5. Bacteria Adherence Assay

*S. pneumoniae* capsule properties were assessed using a mucoviscosity assay adapted from Walker, K.A. et al., 2019 [29,33]. Briefly, *S. pneumoniae* strains were grown to approximately 0.20–0.40 OD_620_ in BHI with MnCl_2_ and CaCl_2_ as indicated and harvested by centrifugation at 4 °C. Cell pellets were resuspended in 1/10 (WT strain) or 1/30 (Δ*mntE* strain) the original culture volume with cold PBS pH 7.4. Cell suspensions were then normalized to 1.0 OD_620_/1 mL with cold PBS pH 7.4, centrifuged at 1000× *g* for 3 min at 4 °C, and the OD_620_ of supernatant was measured. The fraction of cells remaining in solution was calculated by dividing the final OD_620_ by the initial OD_620_. Measurements were performed in duplicate for each independent growth condition. We note that *S. pneumoniae* strains expressing thicker capsules do not form tight pellets, and the supernatants therefore have higher absorbance readings [29].

### 2.6. Biofilm Quantification

Biofilm formation on abiotic surfaces was assessed by crystal violet staining similar to published protocols [34,35]. Briefly, *S. pneumoniae* was grown in TH broth to approximately 0.4 OD_620_ and harvested by centrifugation at 4 °C. Cell pellets were resuspended and diluted to 0.04 OD_620_ in 1 mL TH broth, TH/0.2% D-glucose, or TH/0.3% yeast extract. Each media composition was examined with or without 300 µM MnCl_2_ and/or 1 mM CaCl_2_. Equal aliquots of diluted cell suspension and their corresponding media composition were transferred to a sterile 96-well round-bottom plate (Fisher brand, Rockingham County, NH, USA) in triplicate. After 24 h incubation at 37 °C in a 5% CO_2_ atmosphere, plates were washed with ultrapure water, stained with 0.1% crystal violet for 15 min, and air-dried before being solubilized with 95% ethanol. Absorbance was measured at 570 nm. Each media composition was used as a standard negative control for background through the process.

### 2.7. RNA Extraction and Differential Sequence Analysis

Total RNA was extracted using standard protocol from *S. pneumoniae* grown for 3.5 h in BHI with MnCl_2_ and CaCl_2_ as indicated. Total RNA samples devoid of DNA were sent to the Molecular Research Core Facility (Idaho State University, Pocatello) to determine RNA integrity and further preparation. Briefly, rRNA was depleted and cDNA libraries were generated using the NEBNext ultra II directional RNA library kit (New England Biolabs, Ipswich, MA, USA). Libraries were quantified by Qubit and pooled in equimolar concentrations. Sequencing was performed by the Nevada Genomics Center (University of Nevada, Reno) on one NextSeq2000 P2 SE100 (Illumina, San Diego, CA, USA) 200-cycle sequencing run.

Raw sequencing reads from mRNAseq were quality- and adapter-trimmed using Cutadapt V4.9 [36] with a minimum read length cutoff of 20 nucleotides, prior to mapping on the *S. pneumoniae* D39 (RefSeq NC_0085833) genome using Bowtie2 with the very sensitive option. Read counts were tabulated with HTSeq [37], and differential gene expression analysis was performed as described previously using DEseq2 [38]. Genes were defined as differentially expressed if their *p*-value adjusted for multiple testing (*P*_adj_) was <0.05 and transcript level change was 1.5-fold.

### 2.8. Cell Morphology Measurements

Images of *S. pneumoniae* cells grown for 3.5 h in BHI broth with or without MnCl_2_ or CaCl_2_ as indicated were collected using a Leica DM6B upright brightfield microscope (Leica, Deerfield, IL, USA). The lengths and widths of approximately 100–150 cell bodies for each condition were measured using ImageJ software version 1.51m9, Java 1.8.0_101 (64-bit). The number of cell bodies were counted from 50–100 chains to determine average number of cells/chain.

### 2.9. Statistical Analysis

All growth and assays were independently conducted in at least triplicate. Statistical analyses were performed using GraphPad Prism (Graphpad Prism 9 Software, La Jolla, CA, USA). One-way ANOVA and unpaired *t*-tests were selected with 95% confidence interval. Accepted *p*-values are indicated in figures.

## 3. Results

### 3.1. Ca Rescues Mn^2+^-Sensitive S. pneumoniae ΔmntE Growth Phenotype

**Figure 1:**
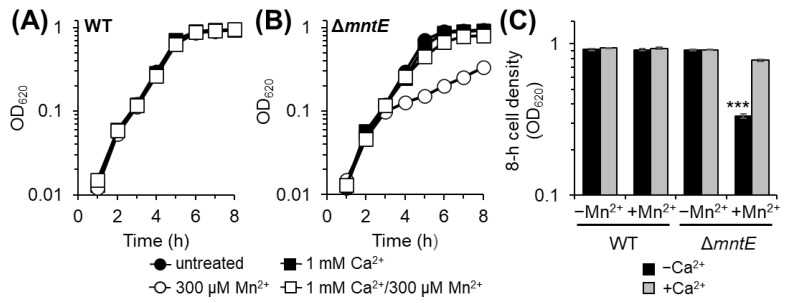
Calcium rescues *S. pneumoniae* Δ*mntE* Mn^2+^-sensitive growth defect. *S. pneumoniae* WT (**A**) and Δ*mntE* (**B**) cultures were diluted to 0.005 OD_620_ at time zero into BHI supplemented with 0 or 300 µM Mn and/or 1 mM Ca^2+^. Turbidity was measured over time at 620 nm. Data shown are the representative growth of at least three independent replicates. (**C**) Mean cell density at 8 h growth of at least three independent replicates ±SEM; ***, *p* ≤ 0.01.

Previous reports demonstrate that *S. pneumoniae* lacking the Mn^2+^-specific CDF efflux transporter MntE (Δ*mntE*) is sensitive to elevated exogenous Mn^2+^ due to its inability to export Mn out of the cell and is thus considered Mn^2+^-stressed [27,30,39]. Likewise, we show here that the *S. pneumoniae* Δ*mntE* strain fails to reach wild-type (WT) cell densities after 8 h when cultured in rich medium with excess exogenous Mn (Figure 1). The addition of 1 mM Ca^2+^ to Mn^2+^-stressed Δ*mntE* permits growth patterns similar to unstressed cells, resulting in the complete rescue of its Mn^2+^-sensitive growth defect (Figure 1B,C). Ca^2+^ alone in the absence of Mn^2+^ stress had no significant effect on the growth of both the WT and Δ*mntE* strains (Figure 1).

**Figure 2:**
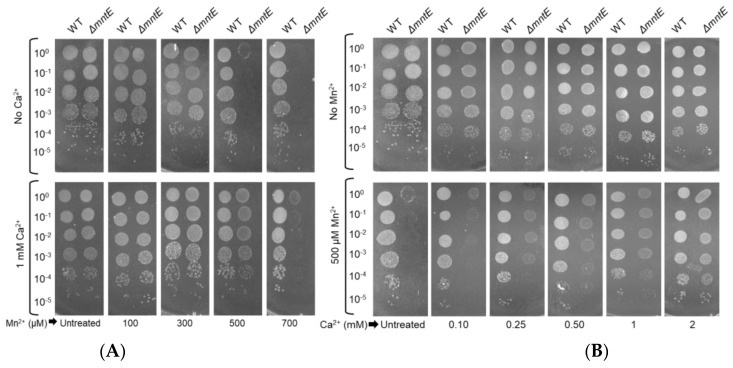
Exogenous Ca^2+^ increases Δ*mntE* strain tolerance to elevated Mn^2+^ levels. (**A**,**B**) *S. pneumoniae* WT and Δ*mntE* cultures were serially diluted and spotted onto BHI/catalase agar supplemented with Ca^2+^ and Mn^2+^ as indicated. Represented growth shown after 24 h incubation of three independent replicates. BHI-only control plate featured is the same in panel (**A**,**B**).

To further evaluate the ability of Ca^2+^ to rescue the Δ*mntE* strain Mn stress growth defect, a serial dilution spot test was performed on rich agar medium supplemented with catalase to prevent hydrogen peroxide intoxication and allow *S. pneumoniae* colonies to form. In comparison to the WT strain, the Δ*mntE* mutant showed significantly diminished growth at 500 µM Mn^2+^ and no growth was observed ≥700 µM Mn (Figure 2A, top). In the presence of 1 mM Ca^2+^, the Δ*mntE* mutant resembled a WT strain growth pattern up to 500 µM Mn^2+^ (Figure 2A, bottom). Residual growth up to 10^−2^ dilution of the Δ*mntE* mutant was observed at 700 µM Mn^2+^ in the presence of Ca^2+^ (Figure 2A).

The Mn concentration was then fixed at 500 µM to examine the minimum Ca^2+^ concentration needed for the recovery of the Δ*mntE* mutant to the WT strain or unstressed Δ*mntE* mutant growth levels. During Mn^2+^ stress, residual growth was observed for the Δ*mntE* strain when Ca^2+^ was supplied between 100 and 500 µM. The growth density of the Δ*mntE* strain rose as the Ca^2+^ levels increased (Figure 2). Complete rescue of Mn^2+^-stressed Δ*mntE* was only visible for Ca^2+^ concentrations ≥1 mM (Figure 2B). These findings are consistent with those observed in broth cultures (Figure 1) and together demonstrate that Ca^2+^ presence is capable of impacting *S. pneumoniae* growth and likely has an intersecting role with Mn metabolism.

### 3.2. Ca^2+^ Rescue of Mn^2+^ Sensitivity Varies among Bacteria

Given the significant impact of Ca^2+^ on *S. pneumoniae* growth during Mn^2+^ stress and the importance of maintaining optimum intracellular Mn^2+^ concentrations among pathogenic bacteria in general, we examined the extent to which Ca^2+^ could rescue other bacteria from Mn^2+^ stress. Two individual bacteria were chosen, *B. subtilis* and *E. coli*, whose Mn^2+^ homeostatic mechanisms including import, export, and their regulation are well characterized [40,41]. We considered *B. subtilis* to be characteristically similar to *S. pneumoniae* in that both are Gram-positive organisms possessing a Mn^2+^-centric physiology, requiring relatively high Mn^2+^ levels for growth compared to other bacteria [30,40,42]. In contrast, *E. coli* is a Gram-negative organism with an iron-centric physiology that conditionally imports Mn^2+^ in response to oxidative stress or Fe^2+^ scarcity [43].

**Figure 3:**
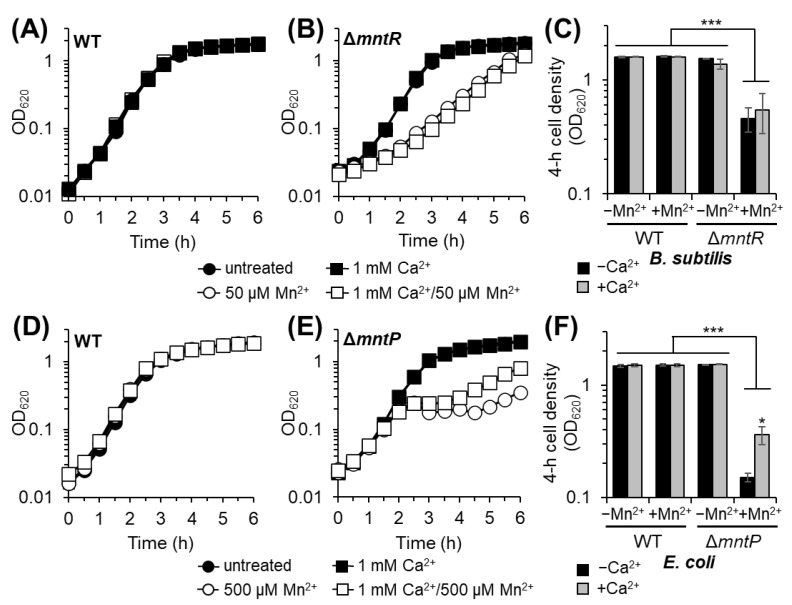
Ca differentially impacts Mn^2+^-sensitive *B. subtilis* Δ*mntR* and *E. coli* Δ*mntP* strains. Exponentially growing cultures were diluted at time zero into BHI with or without 1 mM Ca^2+^ and Mn^2+^ as indicated and turbidity was measured over time at OD_620_. (**A**–**C**) *B. subtilis* strains stressed with 50 µM Mn^2+^. (**D**–**F**) *E. coli* strains stressed with 500 µM Mn^2+^. Growth curves are representative of multiple independent growths. The 4 h cell density is the mean of at least three independent replicates ±SEM; ***, *p* ≤ 0.01; *, *p* ≤ 0.10.

Akin to the *S. pneumoniae* WT strain (Figure 1A), the addition of Ca^2+^ or Mn^2+^ to rich growth medium did not significantly impact the growth of either the *B. subtilis* or *E. coli* WT strains (Figure 3A,D). Consistent with the published literature [41,44], *B. subtilis*, impaired in regulating intracellular Mn^2+^ levels (Δ*mntR*), and *E. coli*, deficient in Mn^2+^ efflux (Δ*mntP*), exhibited reduced growth in the presence of 50 and 500 µM Mn^2+^, respectively (Figure 3B,E). This reduced growth results from Mn^2+^ intracellular accumulation and intoxication [41,44]. The addition of 1 mM Ca^2+^ in the presence of Mn^2+^ stress did not significantly impact the growth of the *B. subtilis* Δ*mntR* strain (Figure 3B,C). We note that Mn^2+^-stressed *B. subtilis* Δ*mntR* cultures reached WT stationary cell density after 6 h incubation, independent of Ca^2+^ presence (Figure 3B). Incubation of *B. subtilis* Δ*mntR* with increased Mn^2+^ (100 µM) enhanced the growth defect, which was not further affected by Ca^2+^ presence (Figure S1).

In contrast to the *B. subtilis* Δ*mntR* strain growth, the addition of 1 mM Ca^2+^ during Mn^2+^ stress partially restored the growth of the *E. coli* Δ*mntP* mutant to half that of the WT strain cell density (Figure 3D,F). Together, these data along with those of *S. pneumoniae* suggest that the roles of Ca^2+^ in relation to cellular Mn^2+^ demand likely vary among bacteria and may instead depend on host environment nutrient conditions and other natural environmental selective pressures.

### 3.3. S. pneumoniae ΔmntE Accumulates High Cellular Mn^2+^ Despite Growth Restoration by Ca^2+^

**Figure 4:**
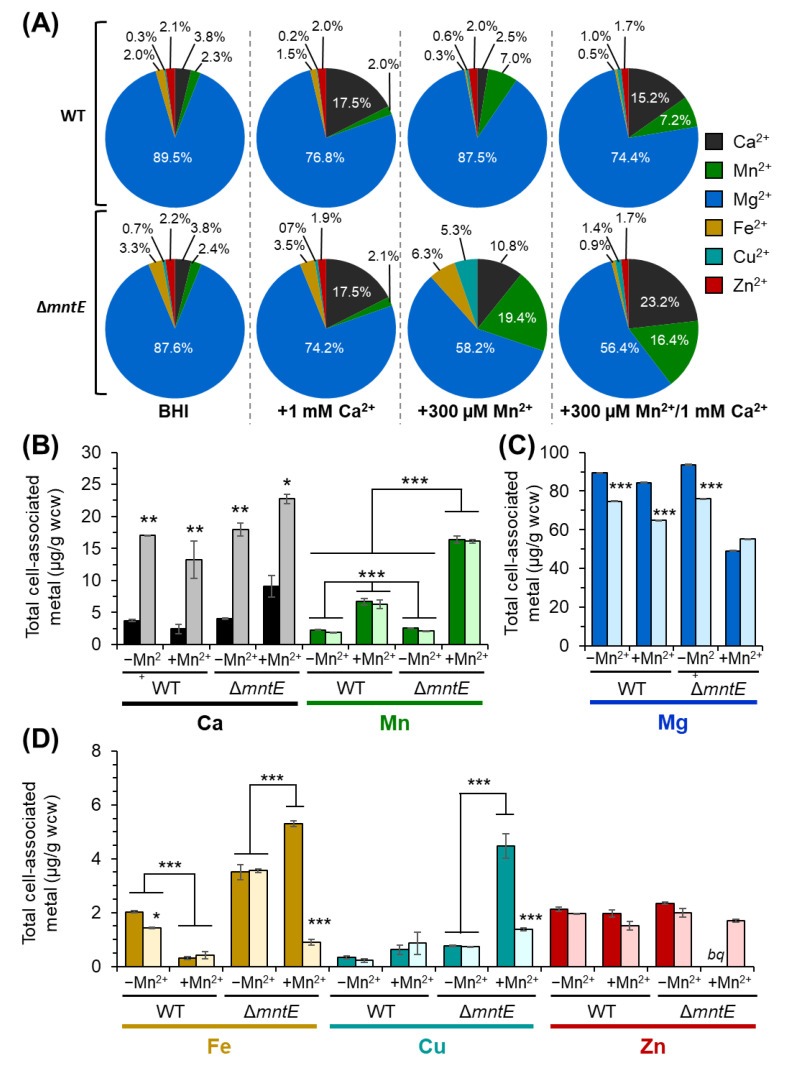
Ca^2+^ enters *S. pneumoniae*, but its presence does not alter cellular Mn^2+^ levels. Total cell-associated metal ion concentrations were measured from *S. pneumoniae* WT and Δ*mntE* grown for 3.5 h with or without 300 µM Mn^2+^ as indicated and 0 (darker shade) or 1 mM (lighter shade) Ca^2+^. (**A**) Metal ion distribution across conditions among for only those metals measured. (**B**) Total cell-associated Ca^2+^ (black) and Mn^2+^ (green); (**C**) Mg^2+^ (blue); (**D**) Fe^2+^ (brown), Cu^2+^ (teal), and Zn^2+^ (red). The mean of at least two independent cultures ±SEM; wcw, wet cell weight. ***, *p* < 0.01; **, *p* < 0.05; *, *p* < 0.10.

To investigate the mechanism by which Ca^2+^ rescues the *S. pneumoniae* Δ*mntE* strain Mn^2+^-sensitive growth phenotype, we next assessed the metal ion composition of bacterial cells. During routine growth in rich medium, the *S. pneumoniae* WT strain cell-associated Ca^2+^ and Mn^2+^ concentration yielded 3.8 ± 0.15 and 2.3 ± 0.07 µg/g wcw (wet cell weight), respectively (Figure 4B). Likewise, similar Ca^2+^ and Mn^2+^ levels were observed for unstressed Δ*mntE* cells, 4.1 ± 0.09 and 2.6 ± 0.06 µg/g wcw, respectively. Cellular Ca^2+^ increased 4.5-fold to 17.0 ± 0.05 and 18.0 ± 1.05 µg/g wcw for *S. pneumoniae* WT and Δ*mntE* strains, respectively, when cultured in the presence of 1 mM Ca^2+^ (Figure 4B), indicating that *S. pneumoniae* is capable of importing Ca^2+^ but the mechanism of how Ca^2+^ enters *S. pneumoniae* is not understood at this time. Incubation with Ca^2+^ alone did not significantly alter other cellular metals examined, including Mn^2+^, zinc (Zn^2+^), iron (Fe^2+^), and copper (Cu^2+^), for both the WT and Δ*mntE* strains (Figure 4A,D) but did slightly reduce magnesium (Mg^2+^) by ≈18% from approximately 89.4 ± 0.45 to 74.8 ± 0.25 µg/g for WT and 93.5 ± 1.00 to 76.0 ± 0.20 µg/g wcw for Δ*mntE* (Figure 4C).

Culturing *S. pneumoniae* WT and Δ*mntE* strains with 300 µM Mn yielded a 3-fold and 8-fold increase in cell-associated Mn for the WT (2.25 ± 0.70 vs. 6.71 ± 0.51 µg/g wcw) and Δ*mntE* (2.56 ± 0.06 vs. 16.4 ± 0.55 µg/g wcw) strains, respectively (Figure 4B). The significant accumulation of cellular Mn^2+^ is consistent with the Δ*mntE* mutant being incapable of efficiently exporting Mn^2+^ out of the cell [27,39]. Interestingly, the Ca^2+^ levels increased 2-fold to 9.1 ± 1.7 µg/g wcw for the Mn^2+^-stressed Δ*mntE* mutant but were relatively unchanged for the WT strain when compared to unstressed cells (Figure 4B). The Δ*mntE* mutant cell-associated Mg^2+^ levels were reduced by almost 2-fold during Mn^2+^ stress, independent of Ca^2+^ presence (Figure 4C). Cell-associated Fe^2+^ and Cu^2+^ rose 1.5-fold (from 3.5 ± 0.28 to 5.3 ± 0.12 µg/g wcw) and 5-fold (from 0.8 ± 0.04 to 4.5 ± 0.46 µg/g wcw), respectively, for the Δ*mntE* mutant during Mn^2+^ stress (Figure 4D), which is consistent with previous reports showing elevated Mn^2+^ interferes with the expression of Fe^2+^ and Cu^2+^ homeostatic proteins [30]. No significant change in Zn^2+^ was observed for the WT strain cultured with Mn (Figure 4D); Zn^2+^ was below the quantifiable detection for the Δ*mntE* mutant. We note here that a high cellular Mn^2+^ concentration can disrupt Zn^2+^ homeostasis in *S. pneumoniae* by possibly binding to the Zn^2+^-sensing regulatory protein SczA and antagonizing its activity to properly regulate the expression of the Zn^2+^-specific CDF exporter CzcD [45] (see Section 3.6 for further support).

When strains were cultured with 1 mM Ca^2+^ during Mn^2+^ stress, the cell-associated Mn^2+^ levels remained elevated for the Δ*mntE* mutant at 16.1 ± 0.30 µg/g wcw (Figure 4B), indicating that the Δ*mntE* strain continues to experience Mn^2+^ intoxication and that exogenous Ca^2+^ is not inhibiting Mn^2+^ import and likely not altering Mn^2+^ export through the secondary Mn^2+^ efflux transporter MgtA [28]. Fe^2+^ and Cu^2+^ were significantly reduced 5-fold and 3-fold, respectively, when Ca^2+^ was added to the Mn^2+^-stressed Δ*mntE* strain (Figure 4D); no significant change in Fe^2+^ or Cu^2+^ was observed for the WT strain.

### 3.4. Ca^2+^ Does Not Alter S. pneumoniae Capsular Polysaccharide Production

To confirm that the *S. pneumonie* Δ*mntE* strain was still physically experiencing Mn^2+^ intoxication despite growth rescue, we next evaluated the production of CPS using two different methods: uronic acid assay (a component of the *S. pneumoniae* D39 bacterial capsule) [32] and mucoviscosity [29,33]. During Mn^2+^ stress, elevated intracellular Mn^2+^ binds to and activates phosphoglucomutase Pgm, a Mn^2+^-requiring enzyme that is critical for the *S. pnuemoniae* biosynthesis of CPS [29]. As such, hyperactivation of phosphoglucomutase Pgm by Mn^2+^ leads to increased CPS production, which prevents *S. pneumoniae* cell adherence to surfaces [29]. For these experiments, the concentration of Mn^2+^ was reduced to 100 µM to ensure growth of the Δ*mntE* mutant over time during Mn^2+^ stress [29,30].

**Figure 5:**
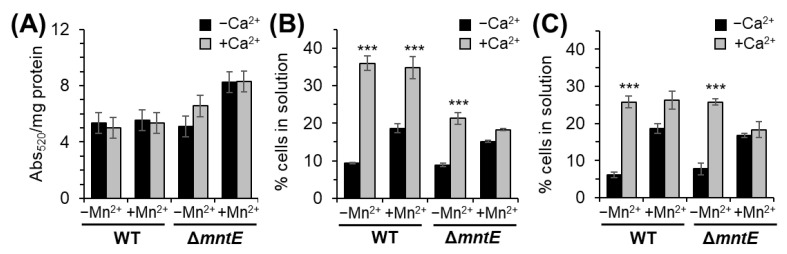
Impact of Ca^2+^ on *S. pneumoniae* CPS production and adherence. (**A**) Direct measurement of the uronic acid, a capsular component, and (**B**) adherence of cells to abiotic surfance grown for 3.5 h with or without 100 µM Mn^2+^ and 2 mM Ca^2+^. (**C**) Adherence of cells to abiotic surface grown with or without 300 µM Mn^2+^ and 1 mM Ca^2+^. We note that *S. pneumoniae* strains expressing thicker capsules do not form tight cell pellets during low-speed centrifugation, and therefore the suspension will have high absorbance. Data shown are the mean of at least three independent cultures ±SEM; ***, *p* ≤ 0.01.

Direct measurement of uronic acid revealed the WT strain CPS production was not significantly altered by Ca^2+^ or Mn^2+^ (Figure 5A). Compared to the WT strain, the unstressed Δ*mntE* mutant produced 24% more uronic acid (Figure 5A). The Δ*mntE* strain CPS levels increased an additional 26% during Mn^2+^ stress, producing ≈45% more uronic acid than WT (Figure 5A). The addition of Ca^2+^ to Mn^2+^-stressed Δ*mntE* did not significantly affect CPS production. These data are consistent with elevated cell-associated Mn^2+^ (Figure 5B) and suggest that cellular Mn^2+^ actively binds to and activates Pgm, leading to thicker CPS.

Qualitative assessment of CPS thickness using mucoviscosity assay during routine growth in rich medium revealed similar mucoviscosity levels for both the WT and Δ*mntE* strains (Figure 5B), which are consistent with those observed by McFarland et al., 2021 [29]. Upon Mn^2+^ exposure, both the WT and Δ*mntE* strains showed a significant increase in likelihood of remaining in solution, up to 2-fold compared to unstressed conditions (Figure 5B). Culturing with Ca^2+^ also reduced cell adherence ability; WT and Δ*mntE* cells were 4- and 2-fold less likely to adhere to the surface compared to unstressed cells, respectively (Figure 5B). Similar adherence levels were observed in the presence of Ca^2+^ during Mn^2+^ stress. We note that Ca^2+^-treated cells appeared “fluffy” and that the cell pellets were easily disrupted if care was not taken. Decreasing exogenous Ca^2+^ did not change these observations and had no significant effect on *S. pneumoniae* cells’ adherence ability (Figure 5C). Together, these data demonstrate that exogenous Ca^2+^ does not influence CPS production in *S. pneumoniae* D39 but may play a role in overall extracellular bacterial surface charge possibly via the expression of adhesion molecules, molecular binding determinants of adhesion molecules, or by other unknown mechanisms.

### 3.5. Mn^2+^ Stress Inhibits S. pneumoniae Biofilm Formation

**Figure 6:**
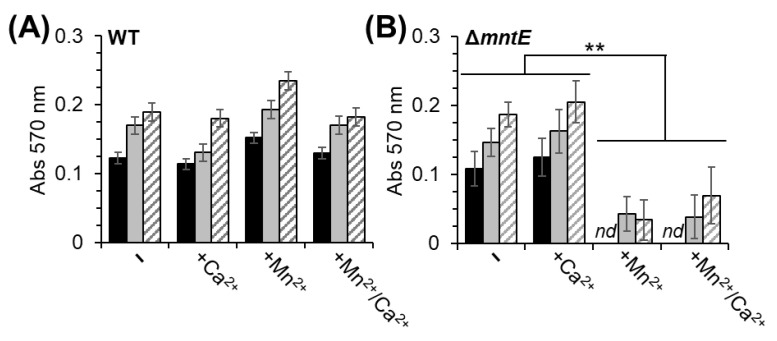
Mn^2+^ stress inhibits *S. pneumoniae* Δ*mntE* biofilm formation, while Ca^2+^ does not. (**A**) WT and (**B**) Δ*mntE* cells were grown in Todd Hewitt (TH) broth with or without 300 µM Mn^2+^ and 1 mM Ca^2+^ for 24 h prior to determining biofilm mass by crystal violet staining. Cultures supplemented with 0.2% glucose (Glc) or 0.3% yeast extract (YE) enhanced relative biofilm formation. Data shown are the mean of at least four independent cultures in triplicate ±SEM. nd, not detected; **, *p* ≤ 0.05.

Like many other upper respiratory tract bacterial pathogens, *S. pneumoniae* forms a biofilm during asymptomatic carriage [46]. These biofilms can increase persistence in the host, as well as *S. pneumoniae* spread among the host population. Furthermore, initial colonization, biofilm formation, and dispersion of *S. pneumoniae* cells from the infection site are each influenced by multiple competing factors and environmental resources, including host nutritional immunity and viral co-infection [47,48]. Since changes in host Ca^2+^ levels have been shown to facilitate bacterial adherence [16,17] and cellular Mn^2+^ influences CPS production [29], we investigated the effect of Ca^2+^ and Mn^2+^ on biofilm formation. For these studies, *S. pneumoniae* was cultivated in TH broth as an alternative to BHI because of its ability to induce consistent biofilm formation [35]. The WT and Δ*mntE* strains showed no significant difference in biofilm formation when cultured in TH broth for 24 h (Figure 6). The addition of Ca^2+^ also did not significantly affect the biofilm formation of either strain. The WT strain biofilm formation was also not affected by Mn^2+^ or the combination of Mn^2+^/Ca^2+^ (Figure 6A). No detectable biofilm formation was observed for the Δ*mntE* mutant during Mn^2+^ stress independent of Ca^2+^ presence (Figure 6B).

We note that other host environmental nutrient factors, including sugar carbohydrates, can also effectively facilitate bacterial biofilm formation [34]. Sugar carbohydrate effectiveness is dependent on its concentration and the bacteria type, as well as other associated factors [49]. Glucose can inhibit initial colonization and early-stage bacterial biofilm formation but can also increase biofilm thickness at later stages of growth [50,51]. We found that supplementation of TH broth with 0.2% glucose or 0.3% yeast extract slightly increased biofilm formation by ≈20% and ≈34% for all conditions examined using the WT strain (Figure 6A); the Δ*mntE* mutant showed similar pattern increases (Figure 6B). Together, these data suggest that carbohydrates and Mn^2+^, but not Ca^2+^ have a role in *S. pneumoniae* biofilm formation. Additional studies are underway to understand the mechanism of how Mn^2+^ modulates biofilm formation.

### 3.6. Ca^2+^ Does Not Alter S. pneumoniae Gene Expression

Having eliminated a direct involvement of Ca^2+^ in modulating *S. pneumoniae* Mn^2+^ transport mechanisms, we sought to gain insight into the mechanism by which Ca^2+^ rescues the Mn^2+^-sensitive Δ*mntE* strain growth phenotype (Figure 1). Since Ca^2+^ is implicated in several sensor pathways in other pathogenic bacteria that regulate genes encoding virulence and resistance proteins [10], we hypothesized that Ca^2+^ might function as a signaling molecule by directly aiding the modulation of *S. pneumoniae* gene expression or by altering cellular protein activities by coordinating with EF-hand motifs found in calmodulin-like proteins that require Mn^2+^.

**Figure 7:**
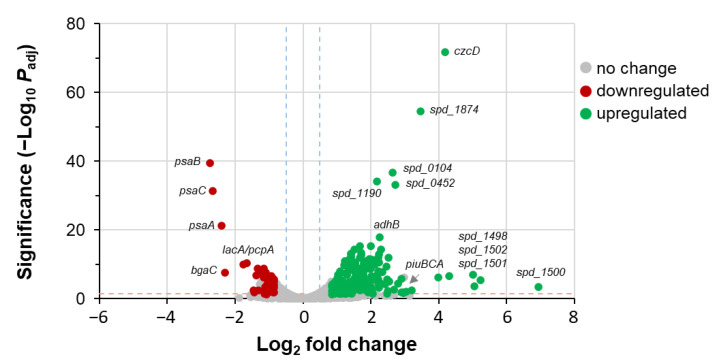
Volcano plot illustrating RNAseq analysis of differentially expressed genes in *S. pneumoniae* during Mn^2+^ stress. Comparison of WT strain cultured in BHI and Δ*mntE* mutant cultured in BHI plus 300 µM Mn^2+^. Genes showing significant difference (*p* ≤ 0.05; dashed blue lines) of ≥1.5 log2 fold change (dashed pink line) are shown in red (downregulated) and in green (upregulated); no significant changes are shown in grey. For a complete list of differentially expressed genes, see Tables S2 and S3.

To investigate this further, differential RNAseq analysis was performed to provide a global snapshot of the relative transcript levels of genes during *S. pneumoniae* growth. Comparison of the transcriptome expression profiles of the WT and Mn^2+^-stressed Δ*mntE* mutant yielded data (Figure 7) consistent with previous reports [30,42]. A total of 273 genes were significantly differentially expressed; there were 65 downregulated (Table S2) and 208 upregulated genes (Table S3). Our data show that during Mn^2+^ stress, the *psaBCA* genes encoding the Mn^2+^-specific ABC-type importer PsaBCA are downregulated 8- to 11-fold. The *czcD* gene encoding the Zn^2+^-specific CDF efflux transporter CzcD is upregulated 5-fold. The latter observations likely result from the mismetallation of the Zn^2+^ regulatory proteins AdcR and SczA by the accumulation of excess cellular Mn^2+^ [45]. *piuBCAD* encoding the Fe^2+^ ABC-type transporter PiuBCAD was induced 8-fold, consistent with previous work [30]. *rffD* (spd_0940)-encoding UDP-N-acetyl-D-mannosaminuronic acid dehydrogenase, which functions in producing sugar precursors for CPS biosynthesis, was upregulated 12-fold in addition to other genes involved in sugar transport and metabolism, which is reflective of the observed increased CPS produced by *S. pneumoniae* during Mn^2+^ stress (Figure 5). Despite observations of a 4.5-fold elevated cell-associated Ca^2+^ (Figure 4A), no significant difference in gene expression was measured in Mn^2+^-stressed Δ*mntE* cultured with and without 1 mM Ca^2+^. Several possibilities are discussed later.

### 3.7. Mn^2+^ Impacts S. pneumoniae Cell Division, Which Is Rescued by Ca^2+^

**Figure 8:**
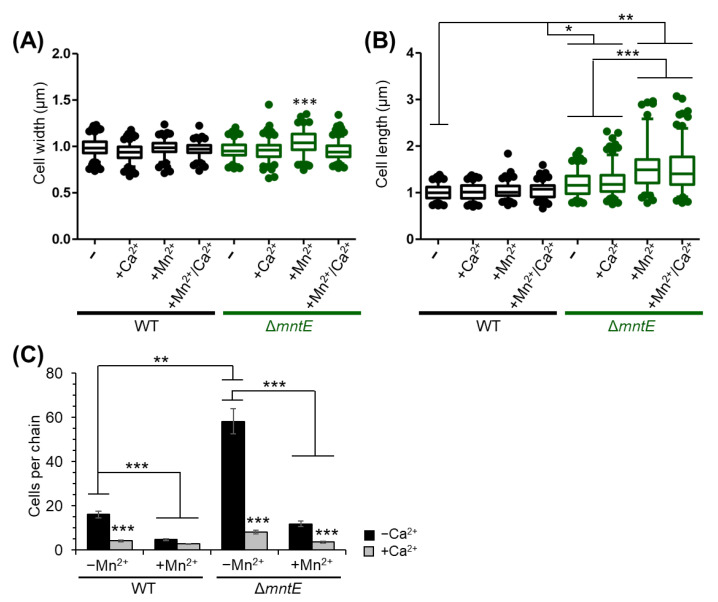
Cell morphology and chain length of *S. pneumoniae* strains stressed for 3.5 h. Width (**A**) and length (**B**) measurements of cells grown with or without 300 µM Mn^2+^ and 1 mM Ca^2+^ as indicated. Box and whisker plot with 95% confidence interval; WT strain shown as black and Δ*mntE* strain in green. (**C**) Mean number of cells observed per chain ±SEM; ***, *p* ≤ 0.001; **, *p* ≤ 0.05; and *, *p* ≤ 0.01.

Accumulation of cellular Mn^2+^ can lead to dysregulation of *S. pneumoniae* cell division via hyperactivation of the Mn^2+^-dependent protein phosphatase PhpP [30]. Such *S. pneumoniae* cells show on average elongated cell bodies, some cell lysis, and increased chaining [30]. We report similar findings here in Figure 8. The average WT cell width and length was 0.99 ± 0.09 and 1.01 ± 0.16, respectively. In general, the WT cell widths and lengths were not significantly changed by Ca^2+^ and Mn^2+^ presence. The Δ*mntE* cells measured slightly wider than the WT cells during Mn^2+^ intoxication at 1.05 ± 0.12 µm vs. 0.98 ± 0.08 µm, respectively (Figure 8A). The addition of Ca^2+^ to the Mn^2+^-stressed Δ*mntE* mutant returned cell widths to the WT or unstressed Δ*mntE* cell sizes.

During routine growth in rich medium, Δ*mntE* cells were on average slightly more elongated than WT cells (1.01 ± 0.16 µm vs. 1.20 ± 0.26 µm for Δ*mntE*). The Δ*mntE* cell lengths significantly increased to 1.54 ± 0.46 µm when cultured with Mn^2+^. The increase appeared to be independent of Ca^2+^ presence, although the median cell length for Δ*mntE* grown with Mn^2+^/Ca^2+^ was lower than with Mn^2+^ alone and was approaching closer to the unstressed Δ*mntE* average cell lengths (Figure 8B). Chaining was also most prevalent for the Δ*mntE* mutant, with an average of 58 cells per chain observed compared to 16 for the WT strain during routine growth (Figure 8C). The addition of Mn^2+^ to the Δ*mntE* mutant resulted in significantly less chaining likely due to increased cell lysis and its reduced ability to divide when toxified by Mn^2+^ [30]. In all cases, the addition of Ca^2+^ led to a significant decrease in chain length; cells were mostly found in pairs or short chains of ≤8 cells per chain depending on the strain (Figure 8C). Cell lysis was not observed microscopically during Ca^2+^ treatment. Taken together with the above findings, the data suggest that Ca^2+^ likely has a role in cell division impacting Mn^2+^ physiology. Further investigation is underway to determine the actual mechanism.

## 4. Discussion

Unlike in eukaryotes, the role of Ca^2+^ in bacteria physiology and virulence remains elusive. Past studies are mostly correlative and have collectively suggested that bacteria may utilize Ca^2+^ as an intracellular signaling messenger whose presence or absence impacts multiple cellular processes, including biofilm formation [10,52]. The present study brings evidence for the involvement of Ca^2+^ in cellular processes requiring Mn^2+^ in *S. pneumoniae* D39. Our data demonstrate for the first time that Ca^2+^ is capable of rescuing *S. pneumoniae* Δ*mntE* Mn^2+^-sensitive growth phenotype via an unidentified mechanism that does not involve altering cell-associated Mn^2+^ levels or gene expression. We further demonstrate that *S. pneumoniae* D39 cells experiencing Mn^2+^ stress produce thicker capsules, which not only inhibit *S. pneumoniae* D39 adhesion to abiotic surfaces but also biofilm formation.

The capacity for Ca^2+^ to restore growth during Mn^2+^ intoxication likely varies among bacteria, since Ca^2+^ supplementation showed some efficacy in *E. coli* but did not impact *B. subtilis* growth. Although more studies are needed to fully access the breadth of Ca^2+^ impact across bacterial pathogens, it is suspected that such differences arise from selective pressures imposed by the environmental niches for which each bacterium resides. Like Mn^2+^ and other metal ions, Ca^2+^ concentrations vary among host sites. Samples taken from the upper respiratory tract and oral cavity can reach millimolar Ca^2+^ levels, with a 10-fold difference between several sites [10]. Furthermore, elevated cellular Ca can trigger the activation of host immune responses against invading pathogens [14,53]. It has been proposed that host Ca^2+^ levels might (1) signal invading bacterial pathogens that they are entering a specific host site and (2) indicate the status of the host immune protection [10]. For example, during bacteria invasion, the EF hand containing S100 proteins like calprotectin require Ca^2+^ to interact with their targets [54]. Activated calprotectin has been shown to sequester Zn^2+^, and subsequently reduces Zn^2+^ interaction with the solute-binding proteins PsaA and MntC in *S. pneumoniae* and *Staphylococcus aureus*, respectively [55]. Sequestration of Zn^2+^ ultimately facilitates Mn^2+^ binding to the solute-binding proteins, resulting in Mn^2+^ import by bacteria. As such, it is likely advantageous for invading bacterial pathogens to recognize host cellular Ca^2+^ levels, which would predictably enhance bacterial adaptation to various host environments, leading to increased bacterial virulence and survival within the host.

Following the revelation that Ca^2+^ rescues Mn^2+^-stressed cells is not mediated through modulation of Mn^2+^ homeostatic proteins, we sought to investigate the molecular mechanism driving the growth rescue. We were somewhat surprised to find that differential RNAseq failed to report any significant changes in global gene expression patterns for the strains grown with or without 1 mM Ca^2+^ during Mn^2+^ stress. It is possible that the exposure time with Ca^2+^ relative to Mn^2+^ stress was not sufficient to observe transcriptional response differences or the relative Ca^2+^ cellular concentration remained within the unknown optimum Ca^2+^ range for *S. pneumoniae* growth. As such, Ca^2+^-specific transcriptional regulatory proteins would likely not be activated to alter gene expression. Note that increasing Ca^2+^, as with other metals studies, also increases the risk of contaminating metals, which may obscure results. Furthermore, the addition of Ca^2+^ concentrations >2 mM in the presence of high Mn^2+^ resulted in precipitation.

Given that Ca^2+^ is not characteristically similar to Mn^2+^ and that Ca^2+^ most often functions as a non-catalytic cofactor in proteins, we speculate that Ca^2+^-mediated rescue in *S. pneumoniae* occurs at the post-translational level. As such, Ca^2+^ would function as a signaling messenger that interacts with cellular proteins to modulate their activity or downstream activity of enzyme targets via post-translational modification or protein stabilization. This is not unprecedented, as several Ca^2+^-sensing two-component regulatory systems have been characterized in other bacterial pathogens [10]. There is also growing evidence showing such novel homeostatic mechanisms for other metal ions including Mn^2+^ [23], which further establishes that metals play important intricate roles in bacteria.

As previously mentioned, CPS production is a critical virulence determinant for many bacterial pathogens, including *S. pneumoniae,* and its production is regulated in response to environmental factors. Mn^2+^ has most recently emerged as an important regulatory component in CPS production for *S. pneumoniae* via the modulation of Pgm activity [29]. Our data are consistent with those reported by McFarland et al., 2021, in that elevated Mn^2+^ increases CPS production [29]. We extended these findings to *S. pneumoniae* D39 biofilm formation, demonstrating that thicker CPS would inhibit transition from a planktonic to sessile-community state. Although the study here is performed in-vitro, these data do reinforce the importance of Mn^2+^ in *S. pneumoniae* colonization and pathogenesis within the host.

Interestingly, we found that Ca^2+^ did not significantly affect biofilm formation but did influence *S. pneumoniae* D39 adherence to surfaces, independent of CPS thickness. Bacterial adhesion capacity is due in part to electrostatic interactions combined with cell surface structures. Gram-positive bacteria, like *S. pneumoniae*, have long glycan chains extending through the cell wall from the cytoplasmic membrane. These glycan chains confer a negative charge and are structurally hydrophilic in nature [56]. As such, extracellular Ca^2+^ might alter the physiochemical properties of the *S. pneumoniae* cell surface via cationic bridging. Direct interaction of Ca^2+^ with such charged exposed surface antigens or extracellular polymeric substances may alter the overall cell surface charge or electrostatic interactions, thereby impeding cell adhesion to surfaces. Further investigation is needed to determine the underlying mechanisms driving this phenomenon and its implication in *S. pneumoniae* virulence and pathogenesis.

In summary, the findings presented in this study highlight a role for Ca^2+^ in *S. pneumoniae* that is interconnected with Mn^2+^ physiology and its implication in virulence. Unraveling the molecular mechanism(s) surrounding the Ca^2+^ rescue of Mn^2+^ intoxication demonstrates the complex nature of how bacterial pathogens have evolved to survive in the host and possibly even hijack host mechanisms to have a selective growth advantage and evade host defenses. It will be interesting to learn how Ca^2+^ physically functions in *S. pneumoniae*, as well as how *S. pneumoniae* imports, senses, and traffics Ca^2+^. Will the Ca^2+^ trend be like that of other metals and be highly regulated to prevent the intoxication and disruption of cellular processes? Nonetheless, it is critical that we gain a better fundamental understanding of Ca^2+^ in bacterial as it will likely provide insights into the regulation of bacterial pathogenesis and aid the development of targeted therapeutics.

## Data Availability

The original contributions presented in the study are included in the article/Supplementary Material; further inquiries can be directed to the corresponding author.

## Supplementary Materials

The following supporting information can be downloaded at: https://www.mdpi.com/article/10.3390/microorganisms12091810/s1, Figure S1: Ca^2+^ supplementation does not rescue the Mn^2+^-sensitive *B. subtilis* Δ*mntR* growth phenotype; Table S1: bacterial strains used in this study; Table S2: genes showing reduced expression that is greater than 1.5-fold and adjusted *p*-value ≤ 0.05 in *S. pneumoniae* WT vs. Δ*mntE* grown in BHI with 300 µM Mn^2+^; Table S3: genes showing increased expression that is greater than 1.5-fold and adjusted *p*-value ≤ 0.05 in *S. pneumoniae* WT vs. Δ*mntE* grown in BHI with 300 µM Mn^2+^; File S1: differential RNAseq summary.

## References

[1] Clapham D.E. (2007). Calcium signaling. Cell.

[2] Permyakov E.A., Kretsinger R.H. (2009). Cell signaling, beyond cytosolic calcium in eukaryotes. J. Inorg. Biochem..

[3] Straley S.C., Bowmer W.S., Gregor L.B. (1993). Regulation by Ca^2+^ in the *Yersinia* low-Ca^2+^ response. Mol. Microbiol..

[4] Tisa L.S., Adler J. (1995). Cytoplasmic free-Ca^2+^ level rises with repellents and falls with attractants in *Escherichia coli* chemotaxis. Proc. Natl. Acad. Sci. USA.

[5] Sarkisova S., Patrauchan M.A., Berglund D., Nivens D.E., Franklin M.J. (2005). Calcium-induced virulence factors associated with the extracellular matrix of mucoid *Pseudomonas aeruginosa* biofilms. J. Bacteriol..

[6] Hu Y., Lu P., Ye C., Zhang H., Wang Y., Chen W., Yang Z. (2011). Structures of Anabaena calcium-binding protein CcbP: Insights into Ca^2+^ signaling during heterocyst differentiation. J. Biol. Chem..

[7] Rosch J.W., Caparon M.G., Nasher H.J. (2008). Calcium efflux is essential for bacterial survival in the eukaryotic host. Mol. Microbiol..

[8] Amankwah S., Abdella K., Kassa T. (2021). Bacterial Biofilm Destruction: A Focused Review on the Recent Use of Phage-Based Strategies with Other Antibiofilm Agents. Nanotechnol. Sci. Appl..

[9] Rumbaugh K.P., Sauer K. (2020). Biofilm dispersion. Nat. Rev. Microbiol..

[10] King M.M., Yendapally R., Asmat T.M., Nagaraja V. (2020). Calcium Regulation of Bacterial Virulence. Adv. Exp. Med. Biol..

[11] Jairaman A., Prakriya M. (2024). Calcium Signaling in Airway Epithelial Cells: Current Understanding and Implications for Inflammatory Airway Disease. Arterioscler. Thromb. Vasc. Biol..

[12] Bagur R., Hajnóczky G. (2017). Intracellular Ca. Mol. Cell.

[13] Asmat T.M., Lin S.Y., Richards D., Gunderson K. (2011). *Streptococcus pneumoniae* infection of host epithelial cells via polymeric immunoglobulin receptor transiently induces calcium release from intracellular stores. J. Biol. Chem..

[14] Gewirtz A.T., Rao A.S., Merlin D., Madara J.L., Jass J.R. (2000). *Salmonella typhimurium* induces epithelial IL-8 expression via Ca^2+^-mediated activation of the NF-kappaB pathway. J. Clin. Investig..

[15] Pace J., Hayman M.J., Galán J.E. (1993). Signal transduction and invasion of epithelial cells by *S. typhimurium*. Cell.

[16] Rose R.K. (2000). The role of calcium in oral streptococcal aggregation and the implications for biofilm formation and retention. Biochim. Biophys. Acta.

[17] Jung C.J., Zheng Q.H., Shieh H.R., Lin C.L., Hsueh P.R. (2017). AtlA Mediates Extracellular DNA Release, Which Contributes to *Streptococcus mutans* Biofilm Formation in an Experimental Rat Model of Infective Endocarditis. Infect. Immun..

[18] Honsa E.S., Johnson M.D., Rosch J.W. (2013). The roles of transition metals in the physiology and pathogenesis of *Streptococcus pneumoniae*. Front. Cell Infect Microbiol.

[19] Trombe M.C., Clavé C., Manias J.M. (1992). Calcium regulation of growth and differentiation in *Streptococcus pneumoniae*. J. Gen. Microbiol..

[20] Trombe M.C., Rieux V., Baille F. (1994). Mutations which alter the kinetics of calcium transport alter the regulation of competence in *Streptococcus pneumoniae*. J. Bacteriol..

[21] Gangola P., Rosen B.P. (1987). Maintenance of intracellular calcium in *Escherichia coli*. J. Biol. Chem..

[22] Watkins N.J., Knight M.R., Trewavas A.J., Campbell A.K. (1995). Free calcium transients in chemotactic and non-chemotactic strains of *Escherichia coli* determined by using recombinant aequorin. Biochem. J..

[23] Martin J.E., Waters L.S. (2022). Regulation of Bacterial Manganese Homeostasis and Usage during Stress Responses and Pathogenesis. Front. Mol. Biosci..

[24] McDevitt C.A., Ogunniyi A.D., Valkov E., Lawrence M.C., Kobe B., McEwan A.G., Paton J.C. (2011). A molecular mechanism for bacterial susceptibility to zinc. PLoS Pathog..

[25] Kloosterman T.G., Witwicki R.M., van der Kooi-Pol M.M., Bijlsma J.J., Kuipers O.P. (2008). Opposite effects of Mn^2+^ and Zn^2+^ on PsaR-mediated expression of the virulence genes *pcpA*, *prtA*, and *psaBCA* of *Streptococcus pneumoniae*. J. Bacteriol..

[26] Johnston J.W., Briles D.E., Myers L.E., Hollingshead S.K. (2006). Mn^2+^-dependent regulation of multiple genes in *Streptococcus pneumoniae* through PsaR and the resultant impact on virulence. Infect. Immun..

[27] Martin J.E., Giedroc D.P. (2016). Functional determinants of metal ion transport and selectivity in paralogous cation diffusion facilitator transporters CzcD and MntE in *Streptococcus pneumoniae*. J. Bacteriol..

[28] Martin J.E., Le M.T., Bhattarai N., Capdevila D.A., Shen J., Winkler M.E., Giedroc D.P. (2019). A Mn-sensing riboswitch activates expression of a Mn^2+^/Ca^2+^ ATPase transporter in *Streptococcus*. Nucleic Acids Res..

[29] McFarland A.L., Bhattarai N., Joseph M., Winkler M.E., Martin J.E. (2021). Cellular Mn/Zn ratio influences phosphoglucomutase activity and capsule production in *Streptococcus pneumoniae* D39. J. Bacteriol..

[30] Martin J.E., Lisher J.P., Winkler M.E., Giedroc D.P. (2017). Perturbation of manganese metabolism disrupts cell division in *Streptococcus pneumoniae*. Mol. Microbiol..

[31] Jacobsen F.E., Kazmierczak K.M., Lisher J.P., Winkler M.E., Giedroc D.P. (2011). Interplay between manganese and zinc homeostasis in the human pathogen *Streptococcus pneumoniae*. Metallomics.

[32] Filisetti-Cozzi T.M.C.C., Carpita N.C. (1991). Measurement of uronic acids without interference from neutral sugars. Anal. Biochem..

[33] Walker K.A., Miner T.A., Palacios M., Trzilova D., Frederick D.R., Broberg C.A., Sepúlveda V.E., Quinn J.D., Miller V.L. (2019). A *Klebsiella pneumoniae* regulatory mutant has reduced capsule expression but retains hypermucoviscosity. mBio.

[34] Loke M.F., Yadav I., Lim T.K., van der Maarel J.R., Sham L.T., Chow V.T. (2022). SARS-CoV-2 spike protein and mouse coronavirus inhibit biofilm formation by *Streptococcus pneumoniae* and *Staphylococcus aureus*. Int. J. Mol. Sci..

[35] Minami M., Konishi T., Takase H., Makino T. (2017). Shin’iseihaito (Xinyiqingfeitang) suppresses the biofilm formation of *Streptococcus pneumoniae* in vitro. BioMed Res. Int..

[36] Martin M. (2011). Cutadapt removes adapter sequences from high-throughput sequencing reads. EMBnet.J..

[37] Putri G.H., Anders S., Pyl P.T., E Pimanda J., Zanini F. (2022). Analysing high-throughput sequencing data in Python with HTSeq 2.0. Bioinformatics.

[38] Sinha D., Zheng J.J., Tsui H.-C.T., Richardson J.D., De Lay N.R., Winkler M.E. (2020). S1 domain RNA-binding protein CvfD is a new posttranscriptional regulator that mediates cold sensitivity, phosphate transport, and virulence in *Streptococcus pneumoniae* D39. J. Bacteriol..

[39] Rosch J.W., Gao G., Ridout G., Wang Y.D., Tuomanen E.I. (2009). Role of the manganese efflux system *mntE* for signaling and pathogenesis in *Streptococcus pneumoniae*. Mol. Microbiol..

[40] Helmann J.D. (2014). Specificity of metal sensing: Iron and manganese homeostasis in *Bacillus subtilis*. J. Biol. Chem..

[41] Martin J.E., Waters L.S., Storz G. (2015). The *Escherichia coli* small protein MntS and exporter MntP optimize the intracellular concentration of Manganese. PLoS Genet..

[42] Lisher J.P., Giedroc D.P. (2013). Manganese acquisition and homeostasis at the host-pathogen interface. Front. Cell Infect. Microbiol..

[43] Anjem A., Varghese S., Imlay J.A. (2009). Manganese import is a key element of the OxyR response to hydrogen peroxide in *Escherichia coli*. Mol. Microbiol..

[44] Huang X., Shin J.-H., Pinochet-Barros A., Helmann J.D. (2017). *Bacillus subtilis* MntR coordinates the transcriptional regulation of manganese uptake and efflux systems. Mol. Microbiol..

[45] Martin J.E., Lisher J.P., Winkler M.E., Giedroc D.P. (2017). The zinc efflux activator SczA protects *Streptococcus pneumoniae* serotype 2 D39 from intracellular zinc toxicity. Mol. Microbiol..

[46] Weimer K.E., Armbruster C.E., Juneau R.A., Hong W., Pang B., Swords W.E. (2010). Coinfection with *Haemophilus influenzae* promotes pneumococcal biofilm formation during experimental otitis media and impedes the progression of pneumococcal disease. J. Infect. Dis..

[47] Hood M.I., Skaar E.P. (2012). Nutritional immunity: Transition metals at the pathogen-host interface. Nat. Rev. Microbiol..

[48] Rowe H.M., Meliopoulos V.A., Iverson A.R., Bickford J.S., Schrock D., Flury E., Feng Y., Xu Y., Albrecht R.A., Sands J. (2019). Direct interactions with influenza promote bacterial adherence during respiratory infections. Nat. Microbiol..

[49] Garcia-Gonzalo D., Pagán R. (2015). Influence of environmental factors on bacterial biofilm formation in the food industry: A review. Postdoc J..

[50] Martin N., Patel J., Munoz F., Sabino J., Yeo T. (2023). Regulation and role of calcium in cellular senescence. Cell Calcium.

[51] Ravi B., Sanyal S.K., Pandey G.K. (2023). Calcium decoders and their targets: The holy alliance that regulate cellular responses in stress signaling. Adv. Protein Chem. Struct. Biol..

[52] Domínguez D.C., Guragain M., Patrauchan M. (2015). Calcium binding proteins and calcium signaling in prokaryotes. Cell Calcium.

[53] Smith D.J., Yau P., Winstanley C., Cummings S.P., Cooper R.J., Knox A.J., Singh M., Walshaw M.J., Chilvers M.A. (2014). Elevated metal concentrations in the CF airway correlate with cellular injury and disease severity. J. Cyst. Fibros..

[54] Rosen T., Wang K.A., Nolan E.M. (2022). Metal sequestration by S100 proteins in chemically diverse environments. Trends Microbiol..

[55] Rosen T., Magnotti E., Spadafora L., Snyder L.M., Goldfarb D., Nolan E.M. (2022). Zinc sequestration by human calprotectin facilitates manganese binding to the bacterial solute-binding proteins PsaA and MntC. Metallomics.

[56] Zheng S., Franklin M.J., Tang T.H., Gilmore B.F., Cornelius V.J., Song S., O’Toole G.A. (2021). Implication of surface properties, bacterial motility, and hydrodynamic conditions on bacterial surface sensing and their initial adhesion. Front. Bioeng. Biotechnol..

