# Supplementary material for: Calcium Rescues Streptococcus pneumoniae D39 ΔmntE Manganese-Sensitive Growth Phenotype
Source: Microorganisms. 2024 Sep 1;12(9):1810. doi: 10.3390/microorganisms12091810 (PMC11434433; doi:10.3390/microorganisms12091810)
Supplement: Supplementary file 1 [file microorganisms-12-01810-s001.zip › Supplementary Material Figure S1 and Tables S1-3.pdf]

Supplementary Material

Calcium Rescues Streptococcus pneumoniae D39 Δ*mntE* Manganese-Sensitive Growth Phenotype

Reuben Opoku[1], Edgar Carrasco[1], Nicholas R. De Lay[2,3], and Julia E. Martin[1,*]

[1]Department of Biological Sciences, Idaho State University, Pocatello, ID 83209, USA

[2]Department of Microbiology and Molecular Genetics, McGovern Medical School, University of Texas Health Science Center, Houston, TX 77030, USA

[3]MD Anderson Cancer Center UTHealth Graduate School of Biomedical Sciences, University of Texas Health Science Center, Houston, TX 77030, USA

*Correspondence: juliamartin@isu.edu; Tel.: 208-282-1277

This file contains **Supplementary Figure S1 and Tables S1-S3.**

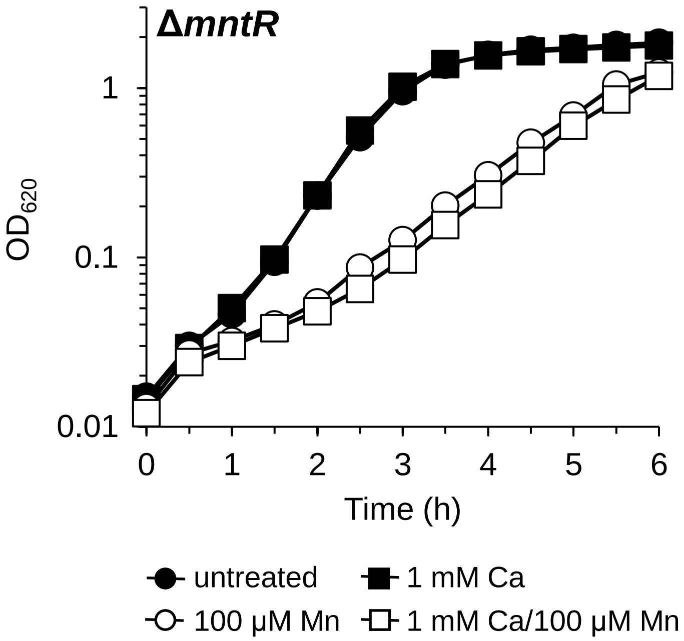

Figure S1. Ca supplementation does not rescue the Mn-sensitive *B. subtilis* Δ*mntR* growth phenotype. Exponentially growing cultures were diluted at time zero into BHI with or without 1 mM Ca and 100 µM Mn as indicated and turbidity was measured over time at OD620. Representative growth curve is shown.

Table S1. Bacterial strains used in this study.

Strain	Genotype	Reference[a]
***Streptococcus pneumoniae* D39**		
IU1781	*rpsL1* (Str^R) wild type	[46]
IU4024	*rpsL1* Δ*mntE* (Str^R)	[21]
Escherichia coli		
MG1655	F$^-$ wild type	JA Imlay
MS025	F$^-$ Δ*mntP::kan*	[28]
***Bacillus subtilis* 168**		
CU1065	*trpC2 att* SPβ	[47]
Δ*mntR*	*rpC2 att* SPβ Δ*mntR*	[47]

[a]References cited here are listed in the main text reference list of associated research article.

Table S2. Genes showing reduced expression that is greater than 1.5-fold and adjusted *P*-value ≤0.05 in *S. pneumoniae* WT vs. Δ*mntE* grown in BHI with 300 μM Mn.

Locus tag	Gene	Known or predicted function(s)	log2 fold change	P_{adj} value
SPD_1461	*psaB*	Mn ABC transporter ATP-binding protein PsaB	-2.7525	2.88E-40
SPD_1462	*psaC*	Mn ABC transporter permease PsaC	-2.6623	4.11E-32
SPD_1463	*psaA*	Mn ABC transporter substrate-binding lipoprotein PsaA	-2.3966	6.19E-22
SPD_0065	*bgaC*	beta-galactosidase	-2.3098	2.81E-08
SPD_1053	*lacA*	galactose-6-phosphate isomerase subunit LacA	-1.7643	1.30E-10
SPD_1965	*pcpA*	choline-binding protein A	-1.6582	5.24E-11
SPD_0096		PadR family transcriptional regulator	-1.4556	3.34E-03
SPD_0095		DUF1700 domain-containing protein	-1.4332	0.015
SPD_0263	*manM*	PTS mannose/fructose/sorbose transporter subunit IIC	-1.3736	1.77E-07
SPD_0262		PTS mannose transporter subunit IID	-1.3395	1.99E-09
SPD_0058	*purD*	phosphoribosylamine--glycine ligase	-1.3239	3.67E-03
SPD_0390	*accA*	acetyl-CoA carboxylase carboxyl transferase subunit alpha	-1.2836	2.09E-08
SPD_0559		PTS fructose transporter subunit IIA	-1.2715	6.98E-05
SPD_1532		PTS beta-glucoside transporter subunit EIIBCA	-1.2697	0.022
SPD_0555		antibiotic ABC transporter permease	-1.2415	1.71E-05
SPD_1052	*lacB*	galactose-6-phosphate isomerase subunit LacB	-1.2400	8.70E-04
SPD_1588		hypothetical protein, putative hemin importer	-1.2045	0.017
SPD_0264	*manL*	PTS mannose transporter subunit EIIAB	-1.1987	1.71E-03
SPD_0389	*accD*	acetyl-CoA carboxylase carboxyl transferase subunit beta	-1.1985	1.14E-07
SPD_0387	*fabZ*	beta-hydroxyacyl-ACP dehydratase	-1.1605	5.35E-07
SPD_1134	*pyrR*	bifunctional pyrimidine operon transcriptional regulator/uracil phosphoribosyltransferase	-1.1549	6.68E-03
SPD_0506	*pheT*	phenylalanine--tRNA ligase subunit beta	-1.1516	1.45E-09

SPD_0557		hypothetical protein	-1.1495	5.04E-07
SPD_0388	*accC*	acetyl-CoA carboxylase biotin carboxylase subunit	-1.1477	2.30E-08
SPD_0384	*fabG*	beta-ketoacyl-[acyl-carrier-protein] synthase II	-1.1467	6.94E-09
SPD_1300		FAD:protein FMN transferase	-1.1358	0.031
SPD_0383	*fabD*	[acyl-carrier-protein] S-malonyltransferase	-1.1325	3.93E-07
SPD_0684		BioY family transporter	-1.1325	9.49E-03
SPD_1887		tRNA-Ile	-1.1234	0.020
SPD_0556		multidrug ABC transporter permease	-1.1209	5.45E-07
SPD_0554		ABC transporter ATP-binding protein	-1.1103	3.15E-04
SPD_1216	*alaS*	alanine--tRNA ligase	-1.1008	2.17E-08
SPD_1217		UDP-N-acetylenolpyruvoylglucosamine reductase	-1.0821	1.07E-05
SPD_0385	*fabF*	beta-ketoacyl-[acyl-carrier-protein] synthase II	-1.0765	2.24E-07
SPD_1051	*lacC*	tagatose-6-phosphate kinase	-1.0660	0.044
SPD_0671		ABC transporter ATP-binding protein	-1.0648	1.22E-06
SPD_0382	*fabK*	2-nitropropane dioxygenase	-1.0621	6.14E-05
SPD_1133	*pyrB*	aspartate carbamoyltransferase	-1.0323	8.12E-03
SPD_0060	*purK*	5-(carboxyamino)imidazole ribonucleotide synthase	-1.0311	3.70E-04
SPD_0267		NCS2 family permease	-1.0222	4.40E-07
SPD_0386	*accB*	acetyl-CoA carboxylase biotin carboxyl carrier protein subunit	-1.0167	1.27E-06
SPD_0380	*fabH*	3-oxoacyl-ACP synthase III	-1.0141	6.84E-05
SPD_0675		KH domain-containing protein	-1.0085	1.44E-06
SPD_1137		multidrug ABC transporter ATP-binding protein	-1.0045	2.94E-04
SPD_1087	*fhs*	formate--tetrahydrofolate ligase	-1.0040	2.59E-03
SPD_0442	*pyrG*	CTP synthetase	-0.9953	3.72E-07
SPD_0374		C4-dicarboxylate ABC transporter	-0.9936	0.021
SPD_1759	*rpoB*	DNA-directed RNA polymerase subunit beta	-0.9860	6.60E-06
SPD_0379	*fabT*	MarR family transcriptional regulator of fatty acid biosynthesis FabT	-0.9837	4.36E-05
SPD_0378		trans-2-decenoyl-ACP isomerase	-0.9748	9.21E-03
SPD_0504	*pheS*	phenylalanine--tRNA ligase subunit alpha	-0.9686	1.01E-05

SPD_0505		N-acetyltransferase	-0.9678	6.50E-05
SPD_1581		thymidylate synthase	-0.9494	4.44E-05
SPD_0700	*pepN*	Aminopeptidase	-0.9451	2.18E-07
SPD_1758	*rpoC*	DNA-directed RNA polymerase subunit beta'	-0.9436	1.98E-05
SPD_1782	*ksgA*	ribosomal RNA small subunit methyltransferase A	-0.9389	8.35E-03
SPD_0159		hypothetical protein	-0.9288	7.61E-03
SPD_0494	*valS*	valine--tRNA ligase	-0.9246	6.60E-07
SPD_1779		thiamine diphosphokinase	-0.9230	9.77E-05
SPD_0741		ABC transporter permease	-0.9186	6.01E-07
SPD_1908		DNA-binding response regulator	-0.9179	3.86E-06
SPD_1781	*rsgA*	GTPase A	-0.9157	8.97E-04
SPD_1334	*atpC*	ATP synthase epsilon chain	-0.9054	1.32E-04
SPD_0742		ABC transporter permease	-0.9047	1.72E-06
SPD_1426		hypothetical protein	-0.8986	2.73E-06
SPD_1132	*carA*	carbamoyl phosphate synthase small subunit	-0.8782	0.013
SPD_0242		hypothetical protein	-0.8691	9.54E-04
SPD_1472	*ileS*	isoleucine--tRNA ligase	-0.8663	5.54E-05
SPD_1326	*pgm*	phosphoglucomutase	-0.8602	2.64E-06
SPD_0757	*rpsA*	30S ribosomal protein S1	-0.8587	0.013
SPD_0349	*fni*	type 2 isopentenyl-diphosphate delta-isomerase	-0.8561	7.82E-06
SPD_1247	*queA*	tRNA preQ1(34) S-adenosylmethionine ribosyltransferase-isomerase QueA	-0.8519	5.36E-04
SPD_1907		Cupin	-0.8455	8.33E-05

Table S3. Genes showing increased expression that is greater than 1.5-fold and adjusted *P*-value ≤0.05 in *S. pneumoniae* WT vs. Δ*mntE* grown in BHI with 300 μM Mn.

Locus tag	Gene	Known or predicted function(s)	log2 fold change	P_{adj} value
SPD_0577	*zmpB*	zinc metalloprotease ZmpB	0.8429	3.18E-4
SPD_1098		amino acid ABC transporter permease	0.8525	1.14E-04
SPD_1034		noncanonical pyrimidine nucleotidase YjjG family	0.8595	0.046
SPD_0352		DNA-binding response regulator	0.8612	4.53E-03
SPD_1528		ABC transporter ATP-binding protein, Na+ export	0.8666	3.28E-03
SPD_0916	*piaB*	ferrichrome-iron ABC transporter permease PiaB	0.8683	4.44E-03
SPD_0108		bacteriocin ABC transporter ATP-binding protein	0.8702	5.38E-04
SPD_1911	*pstC*	phosphate ABC transporter permease subunit PstC	0.8715	0.021
SPD_2033	*yfiA*	ribosomal subunit interface protein	0.8720	0.016
SPD_0247		glycoside hydrolase family 1 protein	0.8723	6.18E-05
SPD_1603		hypothetical protein	0.8768	2.69E-03
SPD_1910	*pstS*	IS110 family transposase	0.8888	7.12E-03
SPD_0005	*pth*	peptidyl-tRNA hydrolase	0.8979	0.020
SPD_0925		ADP-ribosylglycohydrolase family protein	0.9035	1.93E-03
SPD_1986	*fucI*	L-fucose isomerase	0.9143	0.020
SPD_1080		type II restriction endonuclease	0.9237	1.28E-05
SPD_1408		class I SAM-dependent methyltransferase	0.9247	0.032
SPD_0237	*gldA*	glycerol dehydrogenase	0.9313	2.51E-04
SPD_1177		ABC transporter ATP-binding protein	0.9391	3.88E-03
SPD_0236	*talC*	fructose-bisphosphate aldolase	0.9406	1.55E-03
SPD_1081		hypothetical protein	0.9590	2.02E-03
SPD_1294		DUF1836 domain-containing protein	0.9651	7.20E-06
SPD_0355		hypothetical protein	0.9762	1.17E-04
SPD_1913	*pstB*	phosphate ABC transporter ATP-binding protein	0.9834	0.015
SPD_1163		N-acetylneuraminate lyase	0.9865	1.71E-03
SPD_1679	*msmR*	AraC family transcriptional regulator	0.9896	2.57E-04

SPD_1150	*crcB2*	camphor resistance protein CrcB	1.0035	3.59E-05
SPD_0112		XRE family transcriptional regulator	1.0053	8.20E-03
SPD_1747		type 2 lantibiotic	1.0111	1.75E-03
SPD_1527		ABC transporter permease, Na+ export	1.0124	8.35E-07
SPD_1996		DeoR/GlpR transcriptional regulator	1.0189	2.73E-04
SPD_1383	*mgtA*	cation-translocating P-type ATPase	1.0436	8.73E-08
SPD_1831		PTS cellobiose transporter subunit IIC	1.0634	4.74E-03
SPD_1613	*galT-1*	galactose-1-phosphate uridylyltransferase	1.0659	0.011
SPD_1382		glutathione S-transferase	1.0704	4.22E-08
SPD_1902		ABC transporter ATP-binding protein	1.0804	3.81E-05
SPD_0972		hypothetical protein	1.0808	0.032
SPD_0948		carboxylate--amine ligase	1.0851	0.031
SPD_0527		oxidoreductase Fre	1.0857	1.20E-04
SPD_0919		hypothetical protein	1.0892	0.013
SPD_1079		type II restriction endonuclease	1.0903	8.90E-09
SPD_1748		type 2 lantibiotic	1.0922	5.03E-03
SPD_1933	*malQ*	4-alpha-glucanotransferase	1.0985	6.05E-03
SPD_0981		CYTH domain-containing protein	1.0996	6.80E-03
SPD_1518		MutR-like transcriptional regulator, Rgg family transcriptional regulator	1.1019	8.74E-05
SPD_0950		MFS transporter	1.1126	0.013
SPD_0356	*cbpG*	choline-binding protein G	1.1132	1.06E-03
SPD_0943		hypothetical protein	1.1140	0.023
SPD_0357	*cbpF*	choline-binding protein F	1.1155	2.62E-08
SPD_0185		cardiolipin synthase	1.1185	2.92E-04
SPD_1447		DUF2974 domain-containing protein	1.1204	2.57E-04
SPD_2059		YhgE/Pip domain-containing protein	1.1235	0.011
SPD_0928		hypothetical protein	1.1257	0.022
SPD_0354		DNA alkylation repair protein	1.1263	3.41E-06
SPD_1989		PTS mannose transporter subunit IID	1.1325	0.036
SPD_1121		TIGR00341 family protein	1.1366	3.85E-03

SPD_0853	*lytB*	endo-beta-N-acetylglucosaminidase	1.1545	6.50E-05
SPD_2012	*glpO*	glycerol-3-phosphate dehydrogenase/oxidase	1.1566	4.10E-03
SPD_1617		plasmin and fibronectin-binding protein A	1.1618	3.53E-03
SPD_0914	*rumA-1*	RNA methyltransferase	1.1712	1.92E-05
SPD_1414		oxalate:formate antiporter	1.1717	3.25E-03
SPD_0313		S-ribosylhomocysteinase	1.1751	8.07E-06
SPD_1870		pyrrolidone-carboxylate peptidase	1.2124	4.06E-04
SPD_1900		ABC transporter ATP-binding protein	1.2145	1.43E-10
SPD_1987		Fucolectin	1.2145	0.013
SPD_1504	*nanA*	sialidase A	1.2164	2.64E-05
SPD_0930		XRE family transcriptional regulator	1.2289	0.035
SPD_0179		hypothetical protein	1.2313	7.90E-04
SPD_1099		amino acid ABC transporter ATP-binding protein	1.2365	8.62E-12
SPD_0501	*licT*	transcription antiterminator BglG	1.2375	1.71E-03
SPD_2068		serine protease	1.2470	0.024
SPD_1981		hypothetical protein	1.2573	0.019
SPD_0932		hypothetical protein	1.2648	3.55E-05
SPD_0771	*lacR1*	DeoR/GlpR transcriptional regulator	1.2666	0.041
SPD_1680		bifunctional biotin--[acetyl-CoA-carboxylase] synthetase/biotin operon repressor	1.2700	4.45E-05
SPD_0460	*dnaK*	molecular chaperone DnaK	1.2711	4.15E-04
SPD_1833		PTS lactose/cellobiose transporter subunit IIA	1.2873	1.80E-03
SPD_0927	*nplT*	alpha-glycosidase	1.2911	1.11E-07
SPD_0453	*hsdS*	type I restriction endonuclease subunit S	1.2960	2.17E-08
SPD_0528		amino acid ABC transporter permease	1.2973	1.10E-07
SPD_1173		IS5/IS1182 family transposase	1.2999	1.56E-03
SPD_1252		hypothetical protein	1.3037	1.65E-04
SPD_0459	*grpE*	nucleotide exchange factor GrpE	1.3039	1.87E-03
SPD_1178		S9 family peptidase	1.3119	1.14E-04
SPD_0947		hypothetical protein	1.3209	0.014

SPD_1920		rhomboid family intramembrane serine protease	1.3391	8.30E-10
SPD_0913		DUF1002 domain-containing protein	1.3482	1.20E-05
SPD_1100	*zwf*	glucose-6-phosphate dehydrogenase	1.3496	3.79E-12
SPD_0703		DUF3270 domain-containing protein	1.3497	3.44E-05
SPD_1328	*aatB*	glutamine ABC transporter substrate-binding protein	1.3656	9.28E-10
SPD_1046	*lacG-2*	6-phospho-beta-galactosidase	1.3720	3.15E-03
SPD_1355		hypothetical protein	1.3729	8.30E-05
SPD_1677	*rafE*	sugar ABC transporter substrate-binding protein	1.3758	1.46E-04
SPD_0169	*ribD*	bifunctional diaminohydroxyphosphoribosylaminopyrimidine deaminase/5-amino-6-(5-phosphoribosylamino) uracil reductase	1.3927	1.17E-04
SPD_1995	*fucK*	Rhamnulokinase	1.3955	0.026
SPD_0681		hypothetical protein	1.4054	2.36E-03
SPD_0166	*ribH*	6,7-dimethyl-8-ribityllumazine synthase	1.4064	3.89E-0.3
SPD_1921		5-formyltetrahydrofolate cyclo-ligase	1.4208	1.20E-13
SPD_1922		acetyldiaminopimelate deacetylase	1.4226	7.26E-14
SPD_0622		transcriptional regulator	1.4249	8.28E-13
SPD_1162		ROK family protein	1.4270	6.68E-04
SPD_1592		N-acetyltransferase	1.4289	9.84E-05
SPD_0949		Transferase	1.4376	0.034
SPD_0783		restriction endonuclease subunit S	1.4401	3.04E-03
SPD_1675	*rafG*	carbohydrate ABC transporter permease	1.4407	9.91E-04
SPD_0002	*dnaN*	DNA polymerase III subunit beta	1.4431	9.15E-11
SPD_1165		phosphatidylglycerophosphatase A	1.4592	9.28E-10
SPD_0931		toxin PezT	1.4600	7.20E-06
SPD_1676	*rafF*	sugar ABC transporter permease	1.4710	4.80E-03
SPD_0353		DNA alkylation repair protein	1.4742	1.48E-05
SPD_0895	*hemH*	Ferrochelatase	1.4757	4.76E-03

SPD_1594		transcriptional regulator, Xre family	1.4798	2.71E-05
SPD_1984		SPFH domain-containing protein	1.4832	3.82E-05
SPD_0168	*ribE*	riboflavin synthase	1.4906	2.56E-04
SPD_1932	*malP*	glycogen/starch/alpha-glucan family phosphorylase	1.5140	1.05E-08
SPD_1166		hypothetical protein	1.5196	4.55E-15
SPD_1329		amino acid ABC transporter ATP-binding protein	1.5265	3.37E-11
SPD_1164	*cdd-2*	cytidine deaminase	1.5287	3.51E-05
SPD_0529		amino acid ABC transporter permease	1.5303	3.12E-11
SPD_0553		hypothetical protein	1.5315	1.23E-05
SPD_1122	*dprA*	DNA protecting protein DprA	1.5350	6.05E-07
SPD_1167		ABC transporter ATP-binding protein	1.5417	2.31E-09
SPD_0003		DUF951 domain-containing protein	1.5443	2.02E-06
SPD_0933		hypothetical protein	1.5459	5.67E-07
SPD_0502		PTS beta-glucoside transporter subunit EIIBCA	1.5611	2.21E-08
SPD_0167	*ribB*	bifunctional 3%2C4-dihydroxy-2-butanone-4-phosphate synthase/GTP cyclohydrolase II	1.5909	7.31E-07
SPD_0868		Foldase	1.6032	4.59E-07
SPD_0804		ABC transporter ATP-binding protein	1.6081	3.56E-05
SPD_0657	*acuB*	acetoin utilization protein AcuB	1.6357	1.99E-09
SPD_0080		YSIRK signal domain/LPXTG anchor domain surface protein	1.6490	2.20E-09
SPD_1179		lanthionine synthetase	1.6659	1.10E-07
SPD_0805		ABC transporter permease	1.6736	1.30E-09
SPD_1151		tagatose-6-phosphate kinase	1.6755	3.59E-04
SPD_1357	*aliB*	oligopeptide-binding protein AliB	1.6777	9.16E-13
SPD_1330		amino acid ABC transporter permease	1.6839	4.69E-16
SPD_1377		transcriptional regulator	1.6903	2.09E-04
SPD_1170		ABC transporter substrate-binding protein	1.7098	4.11E-14
SPD_1168		ABC transporter permease	1.7252	1.99E-13

SPD_0844	*celB*	DNA internalization-related competence protein ComEC/Rec2	1.7264	1.73E-08
SPD_0615		amino acid ABC transporter substrate-binding protein	1.7271	3.59E-06
SPD_0843	*celA*	competence protein CelA	1.7662	2.31E-05
SPD_1746		hypothetical protein	1.7690	2.42E-03
SPD_0001	*dnaA*	chromosomal replication initiator protein DnaA	1.7736	1.14E-07
SPD_1753		serine protease	1.7740	2.31E-08
SPD_1678	*aga*	alpha-galactosidase	1.7744	4.77E-07
SPD_0531		ABC transporter	1.7843	1.53E-09
SPD_0623	*thiM*	hydroxyethylthiazole kinase	1.7944	7.74E-11
SPD_1633	*galT-2*	galactose-1-phosphate uridylyltransferase	1.7944	1.58E-06
SPD_1171		ABC transporter ATP-binding protein	1.7981	3.49E-11
SPD_1634	*galK*	Galactokinase	1.8321	7.20E-05
SPD_0631	*thiE-2*	thiamine phosphate synthase	1.8353	2.31E-06
SPD_0530		glutamine ABC transporter substrate-binding protein	1.8547	1.14E-11
SPD_2040		Transposase	1.8606	2.92E-03
SPD_0630		hydroxyethylthiazole kinase	1.8740	3.32E-05
SPD_2069		chromosome segregation protein	1.8901	1.23E-05
SPD_1595		hypothetical protein	1.8939	1.92E-05
SPD_1988		hypothetical protein	1.9007	1.91E-03
SPD_1994	*fucA*	fuculose phosphate aldolase	1.9191	0.015
SPD_1172	*nanE-2*	N-acetylmannosamine-6-phosphate 2-epimerase	1.9428	1.64E-09
SPD_1863	*cglA*	type II/IV secretion system protein	1.9519	2.67E-05
SPD_1169		ABC transporter permease	1.9528	3.40E-12
SPD_0503	*bglA-2*	glycoside hydrolase family 1 protein	1.9904	4.69E-16
SPD_0283		PTS cellobiose transporter subunit IIC	2.0024	1.70E-09
SPD_0617		amino acid ABC transporter permease	2.0179	6.67E-04
SPD_1356		ABC transporter ATP-binding protein	2.0216	4.63E-11

SPD_2043		Amidase	2.0263	2.24E-07
SPD_0625		cobalt ABC transporter permease	2.0302	3.97E-06
SPD_0282		hypothetical protein	2.0386	2.59E-03
SPD_0624	*thiE-1*	thiamine phosphate synthase	2.0427	3.79E-12
SPD_1855		hypothetical protein	2.0509	0.032
SPD_1871		hypothetical protein	2.1257	8.48E-11
SPD_0627		hypothetical protein	2.1507	2.05E-07
SPD_1190		Chlorohydrolase	2.1744	6.17E-35
SPD_0616		amino acid ABC transporter ATP-binding protein	2.1933	8.46E-08
SPD_0626		ABC transporter ATP-binding protein	2.2101	8.17E-09
SPD_0280		PTS sugar transporter subunit IIBC	2.2129	2.14E-06
SPD_0249		N5-2CN10-methylene tetrahydromethanopterin reductase	2.2214	8.78E-13
SPD_0618		amino acid ABC transporter permease	2.2267	4.06E-05
SPD_1750	*wrbA*	flavodoxin family protein	2.2386	6.37E-04
SPD_2007		MFS transporter	2.2410	9.65E-14
SPD_1752		peptidase C39	2.2452	2.49E-11
SPD_1751		membrane protein	2.2585	2.35E-05
SPD_1636	*adhB*	Zn-dependent alcohol dehydrogenase	2.2636	1.39E-18
SPD_1862	*cglB*	competence protein CglB	2.2720	3.43E-05
SPD_1749		type 2 lantipeptide synthetase LanM	2.2937	4.55E-15
SPD_0629	*thiW*	thiamine transporter	2.3451	1.57E-08
SPD_1873		hypothetical protein	2.4264	4.82E-05
SPD_1872		MarR family transcriptional regulator	2.4595	4.50E-10
SPD_0628	*tenA*	thiaminase II	2.4716	2.22E-06
SPD_0281		PTS cellobiose transporter subunit IIA	2.4882	0.038
SPD_1857		hypothetical protein	2.5002	4.73E-03
SPD_1637	*nmlR*	MerR family transcriptional regulator	2.5207	9.16E-13
SPD_1354		hypothetical protein	2.5404	1.10E-05
SPD_0104		aggregation factor/peptidoglycan-binding protein LysM	2.6319	2.34E-37

SPD_1858	*creX*	competence protein ComGF	2.6828	1.86E-03
SPD_0452	*trzA*	Integrase	2.7242	6.06E-34
SPD_0940	*rrfD*	UDP-N-acetyl-D-mannosaminuronic acid dehydrogenase	2.8102	3.23E-05
SPD_1505		hypothetical protein	2.8994	1.63E-06
SPD_1650	*piuC*	iron ABC transporter permease PiuC	2.9064	0.016
SPD_1649	*piuB*	iron ABC transporter permease PiuB	2.9525	0.017
SPD_1652	*piuA*	iron ABC transporter substrate-binding protein PiuA	2.9584	0.027
SPD_1267	*piu*	ABC transporter ATP-binding protein	2.9896	5.13E-07
SPD_1651	*piuD*	iron ABC transporter ATP-binding protein PiuD	3.0449	9.48E-03
SPD_1856		hypothetical protein	3.2118	3.26E-03
SPD_1874		N-acetylmuramidase/peptidoglycan-binding protein LysM	3.4616	3.73E-55
SPD_0938		hypothetical protein	3.9890	5.41E-07
SPD_1638	*czcD*	Zn-cation diffusion facilitator transporter	4.1917	2.28E-72
SPD_1499	*nanB*	Sialidase	4.2985	2.05E-07
SPD_1498		gfo/Idh/MocA family oxidoreductase	5.0178	1.16E-07
SPD_1501		sugar ABC transporter permease	5.0564	2.82E-04
SPD_1502		sugar ABC transporter substrate-binding protein	5.2253	4.23E-06
SPD_1500		carbohydrate ABC transporter permease	6.9441	3.39E-04
